# Characterization of spatial and temporal development of Type I and Type II hair cells in the mouse utricle using new cell-type-specific markers

**DOI:** 10.1242/bio.038083

**Published:** 2018-11-15

**Authors:** Stephen McInturff, Joseph C. Burns, Matthew W. Kelley

**Affiliations:** Laboratory of Cochlear Development, National Institute on Deafness and Other Communication Disorders, National Institutes of Health, Porter Neuroscience Research Center, Bethesda, MD 20892, USA

**Keywords:** Inner ear, Utricle, Hair cell, Atoh1, Striola, Calyx

## Abstract

The utricle of the inner ear, a vestibular sensory structure that mediates perception of linear acceleration, is comprised of two morphologically and physiologically distinct types of mechanosensory hair cells, referred to as Type Is and Type IIs. While these cell types are easily discriminated in an adult utricle, understanding their development has been hampered by a lack of molecular markers that can be used to identify each cell type prior to maturity. Therefore, we collected single hair cells at three different ages and used single cell RNAseq to characterize the transcriptomes of those cells. Analysis of differential gene expression identified *Spp1* as a specific marker for Type I hair cells and *Mapt* and *Anxa4* as specific markers for Type II hair cells. Antibody labeling confirmed the specificity of these markers which were then used to examine the temporal and spatial development of utricular hair cells. While Type I hair cells develop in a gradient that extends across the utricle from posterior-medial to anterior-lateral, Type II hair cells initially develop in the central striolar region and then extend uniformly towards the periphery. Finally, by combining these markers with genetic fate mapping, we demonstrate that over 98% of all Type I hair cells develop prior to birth while over 98% of Type II hair cells develop post-natally. These results are consistent with previous findings suggesting that Type I hair cells develop first and refute the hypothesis that Type II hair cells represent a transitional form between immature and Type I hair cells.

## Abstract (precis)

**Summary:** Single cell RNAseq was used to identify new markers for specific hair cell in the mammalian utricle. With these new markers, the developmental timing of specific types of hair cells was examined.

## INTRODUCTION

**Fig. 1.**
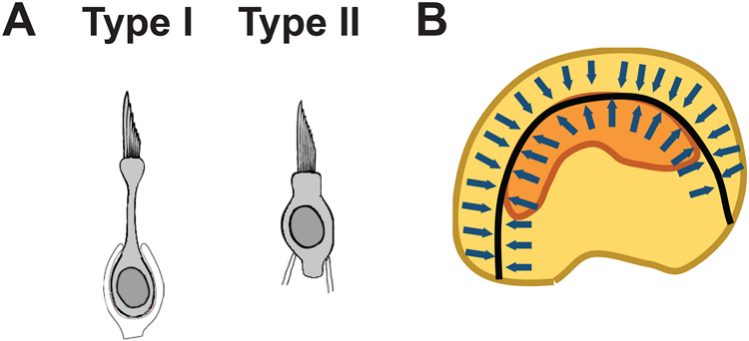
**Morphology of the utricle.** (A) Line drawings illustrating the morphologies of Type I and Type II HCs in the utricle. Type I HCs are flask shaped with a narrow neck while Type II HCs are cylindrical. In addition, Type I HCs are innervated by calyceal endings while Type IIs have bouton like synapses. (B) Overall morphology of the utricle. Arrows indicate the general orientation of HC stereociliary bundles relative to the line of reversal (black). The striola is indicated in orange.

Within the vertebrate inner ear, vestibular sensory structures are stimulated by changes in acceleration or angular velocity facilitating the perception of head position or movement (Burns and Stone, 2017). The two otolithic sensory patches, the utricular and saccular maculae, mediate the perception of linear acceleration while the cristae associated with each of the three semi-circular canals detect angular motion. Each of the vestibular sensory epithelia contains mechanosensory hair cells (HCs) that act as the primary transducers for vestibular signaling. Utricular/saccular HCs clearly differ from HCs found in the cristae in terms of the length of their stereociliary bundles. But even within a single epithelium, HCs can be divided into Type I or Type II based on morphology, physiology and innervation (Deans, 2013; Eatock et al., 1998). In particular, Type I HCs are characterized by the presence of calyceal nerve endings that envelope almost the entire basolateral surface of the HC. In contrast, Type II HCs are contacted by multiple afferent fibers that form bouton-like synapses (Eatock et al., 1998). In addition, there are differences in morphology; Type I HCs are generally flask-shaped with a narrow neck while Type II HCs are more cylindrical (Fig. 1A). Finally, Type I HCs express a low-voltage-activated, outward-rectifying conductance, termed g_K,L_ which produces smaller, faster voltage responses by comparison with the K_V_ conductance in Type II HCs (Eatock and Songer, 2011; Rusch and Eatock, 1996). Despite clear differences in phenotype and function, our understanding of the development of vestibular HC types, including timing and specification remains limited.

Based on several studies, the development of the utricle is known to begin as a patch of sensory epithelium located in the mid-ventral region of the embryonic inner ear. Some cells within the sensory patch become post-mitotic as early as E11 in the mouse (Denman-Johnson and Forge, 1999; Raft et al., 2007; Ruben, 1967) and the first differentiating HCs can be seen by E12. However, unlike the cochlea, the utricular sensory patch continues to expand in size and add HCs through the early post-natal period. The spatial pattern of HC addition is less clear. Most growth occurs at the periphery of the sensory patch but HCs are also added centrally (Burns et al., 2012). Onset of function, based on vestibular-ocular reflex, occurs around P10, but when the epithelium becomes functionally mature is less clear. Further, the developmental timing and determination of Type I and Type II HCs is not known. This is in part because definitive molecular markers for each HC type have not been identified. Therefore, we used a combination of fate mapping and single cell RNA-Seq to identify new markers for Type I and Type II HCs and to map HC type development in the embryonic and post-natal utricle.

## RESULTS

### Spatiotemporal development of utricular HCs

The shape of the adult utricular sensory epithelium is ovoid with a noticeable hilus on the medial side. Further, a crescent-shaped striolar region containing a high density of Type I HCs is located near the center of the epithelium (Li et al., 2008; Desai et al., 2005) (Fig. 1B). There is also a greater density of Type I HCs compared to Type II HCs in the extrastriola, although the ratio is slightly less than in the striola. K_v_ currents of neonatal striolar HCs are slower to activate and less likely to inactivate compared to their extrastriolar counterparts, suggesting there could be further subtype diversity of the major Type I and Type II classes.

While the specific roles of Type I and Type II HCs have not been determined, it is believed that the more derived Type I cells play a role in the perception of faster head movements (Curthoys et al., 2017; Eatock and Songer, 2011; Goldberg et al., 1990). Finally, HC stereociliary bundles are oriented differently in different regions of the utricle. In the medial 2/3s of the utricle, including the striola, HCs are uniformly oriented towards the lateral side while in the lateral 1/3 of the epithelium, cells are oriented medially (Fig. 1B). This pattern creates a line of polarity reversal located just lateral to the striolar region.

**Fig. 2.**
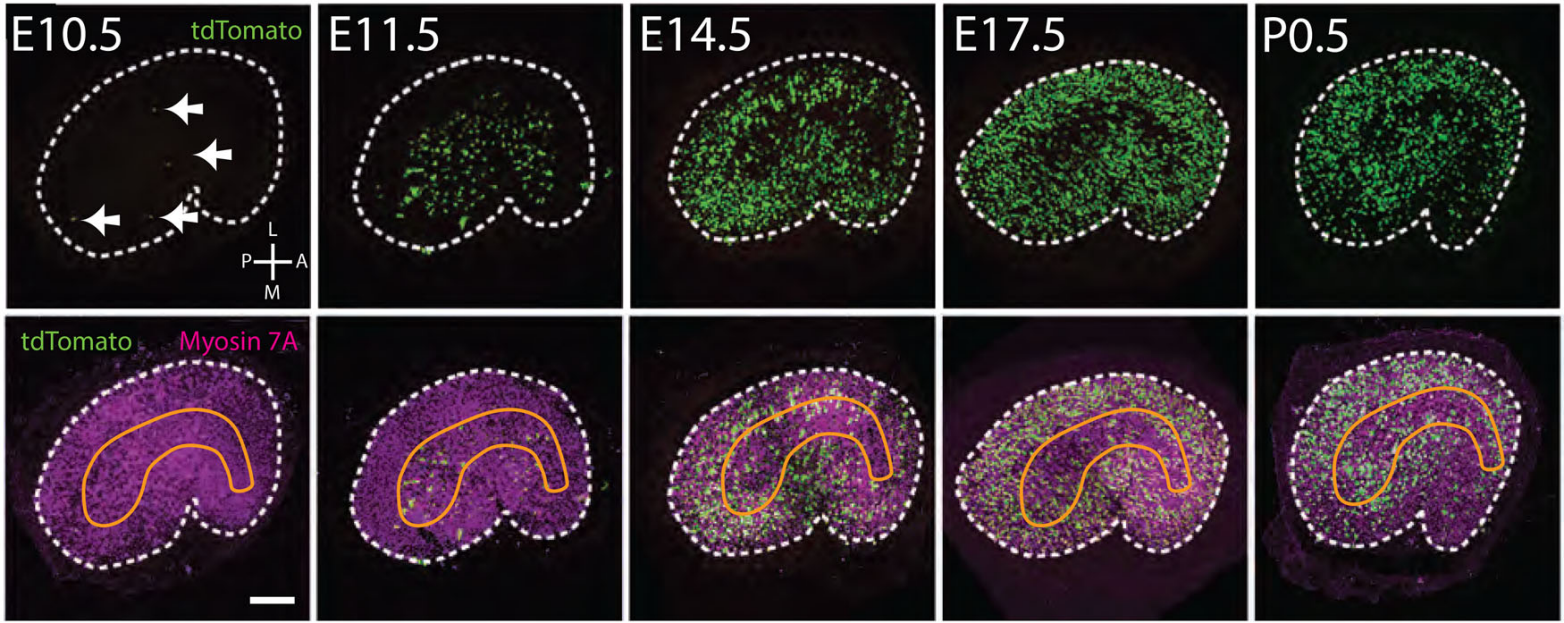
**HCs develop in a central-to-peripheral gradient.** Whole mount images of utricles from adult *Atoh1^creErt2^;R26R^tdTomato^* mice injected with Tamoxifen at the indicated gestational ages. Cells that expressed *Atoh1^+^* on the day of injection are marked in green and all HCs (Myosin7A^+^) are in magenta. Boundaries of the utricular sensory epithelium are indicated by dashed lines in each panel. The approximate position of the striola is indicated in orange in the lower row. The average number of *Atoh1^+^* cells labeled at E10.5 is low (8 per utricle) and those cells are scattered in the central posterior region (arrows). Induction at later time points indicates a central-to-peripheral gradient of addition of HCs. Orientation for all images is indicated in the upper left panel. Scale bar: 100 µm.

To determine the timing and pattern of HC development in the utricle, expression of an early, but transient, HC marker, *Atoh1* (*Atoh1^CreErt2^*) was combined with an *R26R^tdTomato^* reporter to mark new HCs generated at embryonic time points. Labeling was induced by injecting pregnant females with tamoxifen on E10.5, E11.5, E14.5, or E17.5. In addition, newborn pups were injected on P0.5. All animals were maintained until maturity (>P60) prior to fixation. Utricles were dissected and cells that expressed *Atoh1* at the time of induction were identified based on expression of tdTomato. HCs were labeled using an antibody against Myosin7A. Induction on E10.5 labeled a small number of HCs (average of 8 per utricle, *n*=4) which were located in the central region of the utricle, on the medial side of the line of reversal but in both striolar and medial extrastriolar regions (Fig. 2). The number of *Atoh1^+^*-HCs increased by an order of magnitude when induced at E11.5 (average 113.8, *n*=6), however labeled cells were still largely restricted to the central region of the utricle and did not cross the line of reversal (Fig. 2). Induction at E14.5 labeled more than twice as many cells (281.7, *n*=7) as at E11.5 and the distribution of those HCs included the entire central region of the utricle on both sides of the line of reversal. At E17.5, over 1200 *Atoh1^+^*-HCs were labeled with a distribution that included the entire utricle except for the extreme periphery. Finally, induction at P0.5 marked a similar number of HCs as observed at E17.5 with a distribution that extended throughout the sensory epithelium.

### Single cell RNA-Seq analysis of utricular HCs

**Fig. 3.**
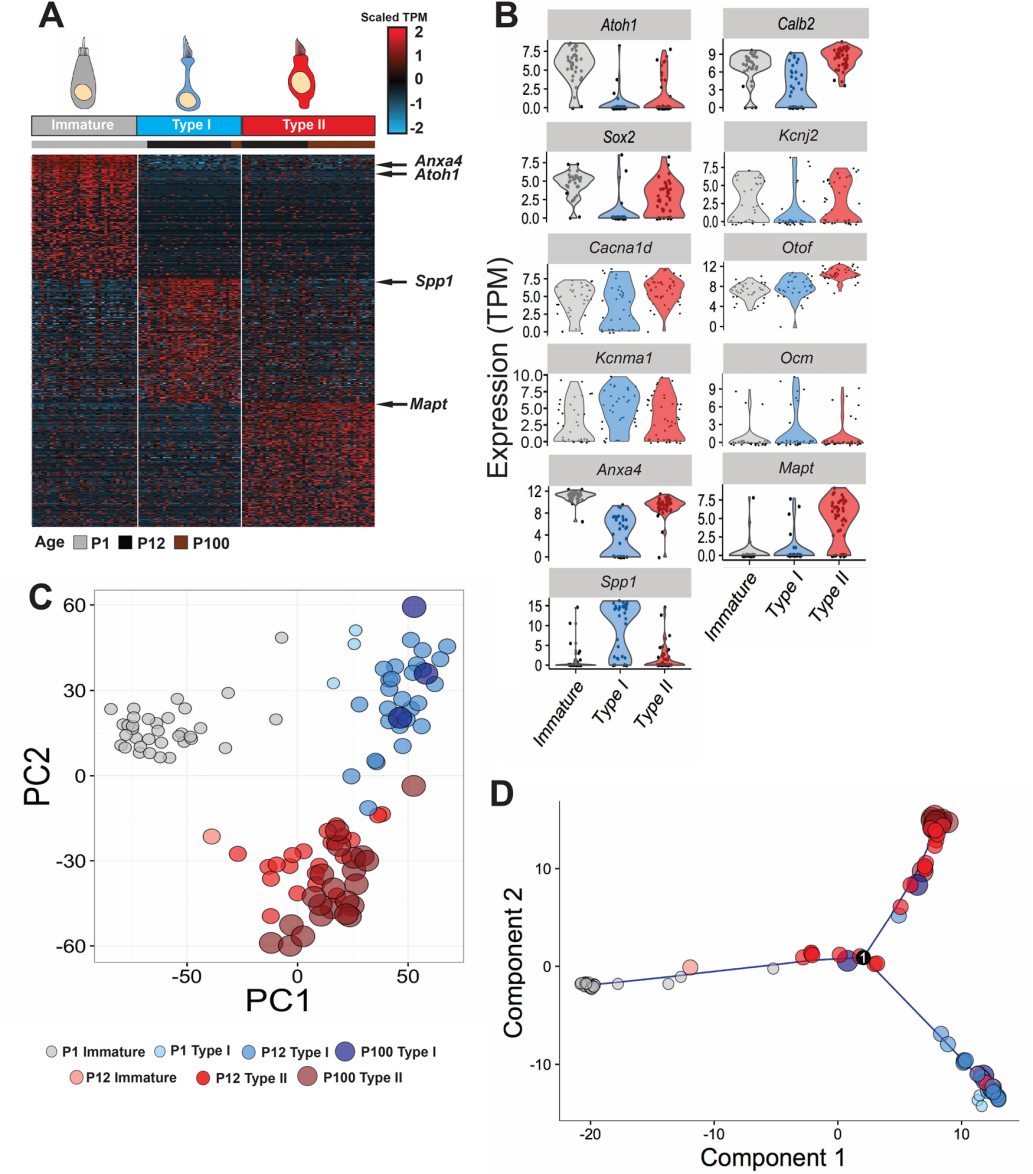
**Single cell analysis of HC development in the utricle.** (A) Heat map depicting expression of the top 200 differentially expressed genes in the single cell data set. Unbiased clustering indicates three distinct groups of HCs. Immature HCs are all derived from P1 utricles while the other two clusters, preliminarily classified as Type I and Type II, contain cells from both the P12 and P100 captures with a limited number of P1 HCs in the Type I group. (B) Violin plots illustrating expression levels for specific genes in cells from each HC group. Known markers of Type II HCs such as *Calb2* and *Kcnj2* show higher expression in the immature and putative Type II groups. In addition, three potential novel markers, *Anxa4* and *Mapt* for Type II HCs and *Spp1* for Type I HCs, were also identified. (C) Principal component analysis for the three groups identified in A. Each circle represents an individual cell and the age and type for each cell is indicated based on color and size. (D) Trajectory analysis for the same cells using Monocle. The results place immature hair cells at one side of the trajectory prior to a branch point that separates cells predominantly into Type I and Type II HC groups.

As discussed, understanding the development of specific subtypes of utricular HCs has been difficult because of a lack of molecular markers that can be used to mark Type I or Type II HCs at ages prior to the maturation of afferent innervation during the first post-natal week (Rusch et al., 1998). To identify new markers for each HC type, single HCs from P12 and P100 utricles were captured using the Fluidigm C1 platform and then profiled by RNA-Seq. A total of 51 HCs were collected at P12 and 25 HCs at P100. These data were then combined with a previously published single cell data set containing 37 P1 utricular HCs (Burns et al., 2015). Unbiased clustering of HCs from the three ages indicated three primary groups of cells. Most P1 HCs clustered together, suggesting that HCs at this stage are largely homogenous and immature (Fig. 3A). Consistent with this conclusion, expression of *Atoh1*, a marker of immature HCs (Chen et al., 2002; Driver et al., 2013), was highest in the P1 cluster (Fig. 3A,B). In contrast, the remaining two groups contained cells from multiple time points. As a first step towards determining the identities of the cells in the other clusters, expression of *Calbindin2* (*Calb2*), also known as *Calretinin*, a known Type II HC marker in adult mice (Li et al., 2008; Desai et al., 2005; Zheng and Gao, 1997) was examined. Results indicated strong expression of *Calb2* in both the P1 cluster and one of the mixed age HC clusters. Based on this pattern of expression, we hypothesized that the mixed age *Calb2^+^* cluster represents Type II HCs. The remaining mixed age HC cluster was tentatively designated as containing Type Is by process of elimination. To visualize the relationships between these cells, Principal Component Analysis (PCA) was performed (Fig. 3C). The three groups of cells identified by unbiased clustering were evident in the plot of the first two PCs with P1 HCs separated from older HCs along PC1. The remaining two clusters, tentatively designated as Type I and Type II were separated along PC2. Interestingly, Type I HCs segregated further along PC1 than did Type II HCs, suggesting that Type IIs might share more similarities with immature HCs.

While these two clusters were primarily comprised of cells from P12 and P100, a small number of cells from the P1 data set clustered with the putative Type I group (smallest circles, light blue in Fig. 3C), suggesting that some mature Type I HCs (approximately 8.5% based on the total number of single cells captured at P1) are present in the utricle at P1. This result is consistent with published findings indicating the presence of a limited number of Type I HCs at P0 (Geleoc et al., 2004; Rusch et al., 1998). In our previous report (Burns et al., 2015) we identified these cells as putative striolar Type I HCs since some cells in the cluster expressed *Ocm*, a known marker of this cell type (Simmons et al., 2010). At the clustering resolution utilized for this analysis, we only detected two major types of HCs. While further sub-clustering of the putative Type I population did suggest the presence of more diversity, our sample sizes were not high enough to draw definitive conclusions. Thus, we hypothesize that the largest separation in identity is between Type I and Type II HCs, independent of region, rather than striolar versus extrastriolar.

The distribution of cells along PC1 and PC2 suggested possible differentiation of immature cells along two paths, one leading to Type I HCs and a second leading to Type II HCs. To determine if this was the case, trajectory analysis was performed using Monocle (Trapnell et al., 2014) (Fig. 3D). Results indicate a trajectory with a single branch point. Immature cells are located at one node while Type I and Type II HCs are predominantly located along the two other branches.

### Identification of new markers for HC subtypes

To determine whether the two clusters comprising cells from different ages represent Type I and Type II HCs, we examined expression of known markers for each cell type in the single cell data set. Expression of *Sox2*, which is broadly expressed in immature HCs but subsequently down-regulated in Type I HCs (Oesterle et al., 2008), was significantly upregulated in the immature and putative Type II clusters (*P*<0.05; Fig. 3A,B). Similarly, *Kcnj2* (*Kir2.1*), which has been reported to be expressed in most immature HCs but is only maintained in Type IIs, showed significant elevation in the immature and putative Type II clusters (Levin and Holt, 2012). Therefore, known markers of Type II HCs were enriched in the immature and Type II clusters.

Next, we examined the expression of four genes, *Cacna1d* (*Cav1.3*), *Otof* (*Otoferlin*), *Kcnma1* (*BK*) and *Ocm* (*Oncomodulin*), with reported expression in Type I HCs. Kcnma1 and Ocm are restricted to striolar Type I HCs, whereas Cacna1d and Otof are thought to be expressed in both extrastriolar and striolar Type I HCs (Bao et al., 2003; Hoffman et al., 2018; Ramakrishnan et al., 2009; Schug et al., 2006; Schweizer et al., 2009; Simmons et al., 2010). Violin plots for each gene indicate expression in all three clusters, although with different levels of expression in each subtype (Fig. 3B). Consistent with striolar Type I HCs making up a small fraction of the total HC population, *Ocm* was expressed in a few cells in all three clusters; however, it did show a limited, but significant increase in the putative Type I cluster (*P*<0.05). *Cacna1d* and *Otof*, which have been reported to be exclusively expressed in Type I HCs at adult ages (Ramakrishnan et al., 2009; Schug et al., 2006), showed strong expression in all three clusters but no significant bias towards Type I HCs. The results confirm known markers of striolar Type I HCs, but reported pan markers of Type I HCs (i.e. extrastriolar and striolar) did not show clear restriction to the putative Type I cluster.

**Fig. 4.**
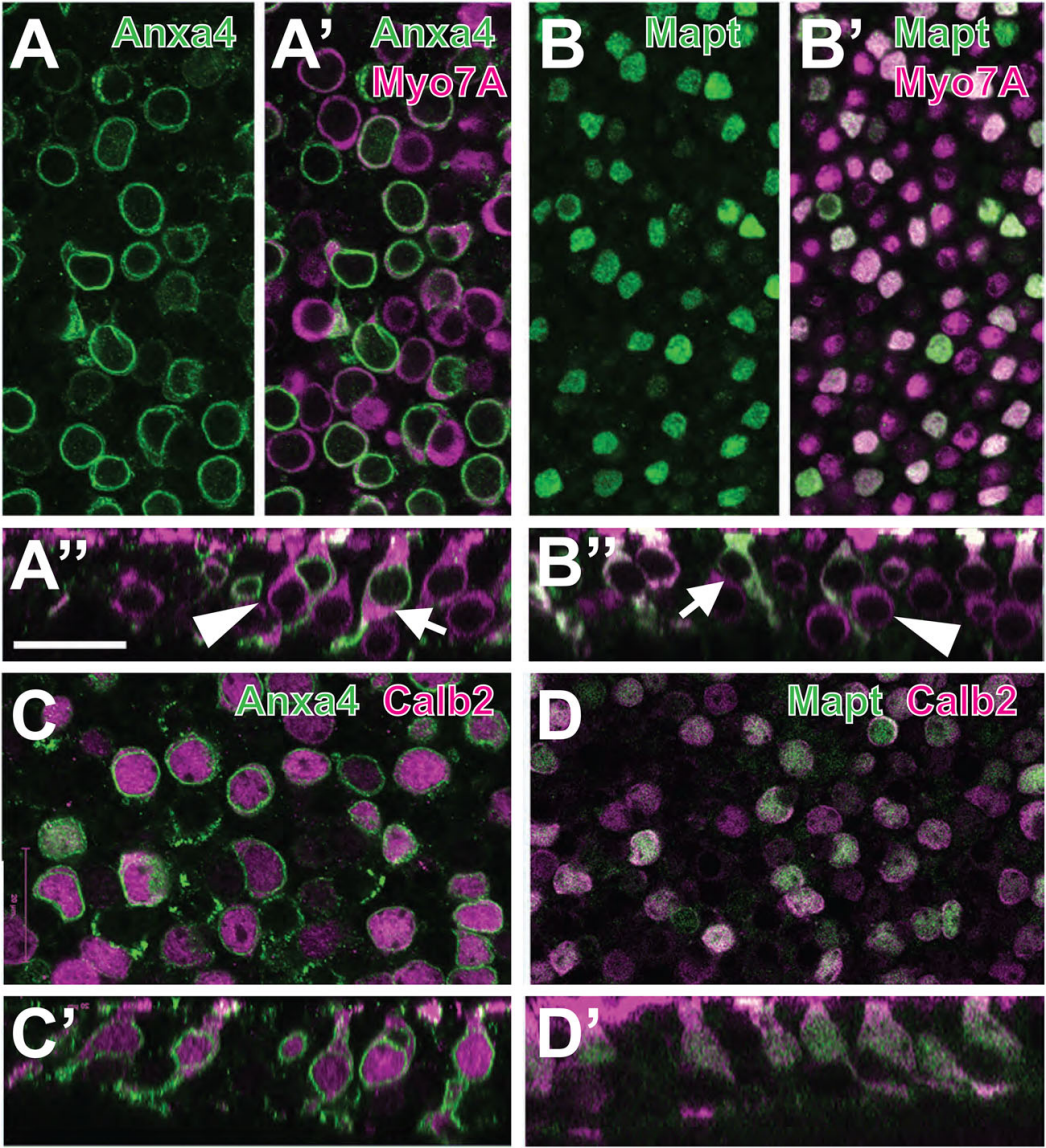
**Anxa4 and Mapt are markers for Type II HCs.** (A) Surface view from a P64 utricle labeled with anti-Anxa4 (green). Anxa4 is expressed along the cell and nuclear membranes of a subset of cells. (A′) The same view as in A, but also showing expression of the pan-HC marker Myo7a in magenta. Anxa4 co-localizes with a subset of HCs. (A″) Orthogonal view from the same sample as in A. Arrow indicates an Anxa4-positive cell. Note the more lumenal position of the nucleus and the wider HC neck. By comparison, an Anxa4-negative cell (arrowhead) has a more basal nucleus and a narrow neck. (B–B″). Similar views as those in A, except with expression of Mapt illustrated in green. Arrow and Arrowhead in B″ illustrate similar differences in the morphologies of Mapt^+^ and Mapt^−^ cells. (C,D) To confirm that Anxa4 and Mapt are markers of Type II HCs, P62 utricles were double-labeled with Anxa4 (C) or Mapt (D) and the Type II HC marker, Calb2. Note co-labeling of both Anxa4 and Mapt with Calb2. Scale bar in A″, same for all other panels: 20 µm.

**Fig. 5.**
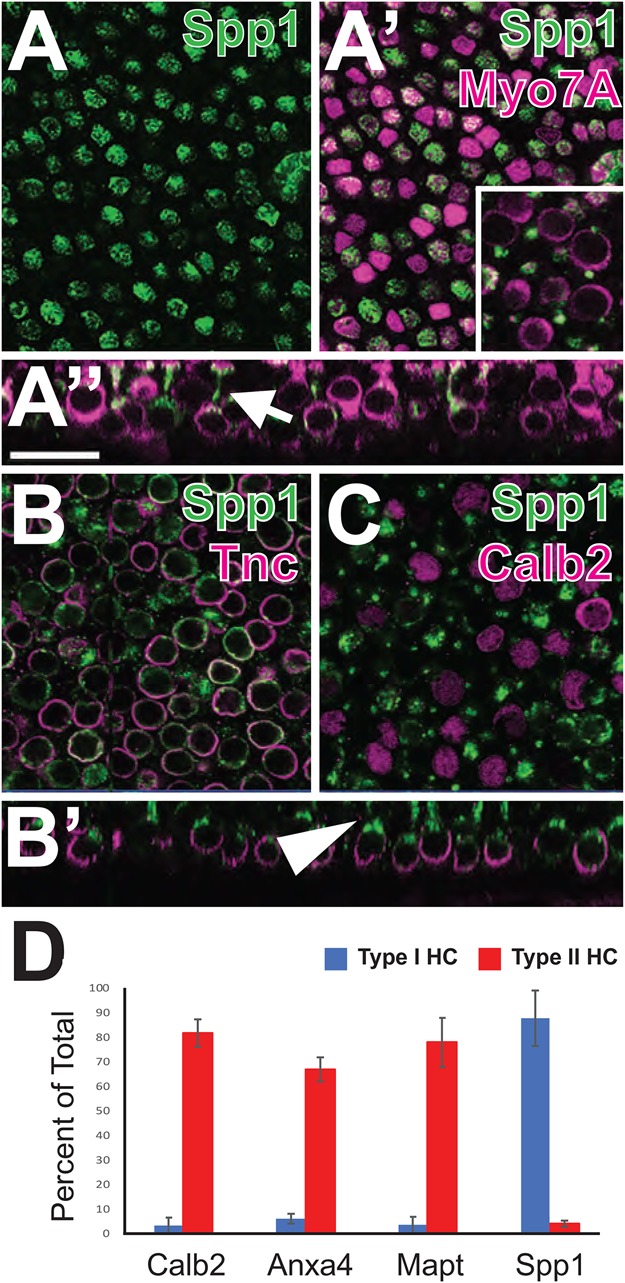
**Spp1 is a marker for Type I HCs.** (A) Surface view of expression of Spp1 (green) in a P70 utricle. (A′) The same view as in A, but with Myo7A labeling (magenta). Spp1 is present in a subset of HCs. Inset: similar view as in A′ but focused on the level of Type II cell bodies. Note that Spp1 is expressed in narrow profiles located in between the Type II cell bodies. (A″) Orthogonal view from the same sample as in A. Spp1 is restricted to the necks of flask shaped HCs (arrow). (B) Spp1^+^ cells are co-labeled with Tnc, a marker of the calyceal endings surrounding the basal sides of Type I HCs. (B′) Orthogonal view from the same sample as in B. Note that Spp1 is restricted to the neck regions of Type I HCs (arrowhead). (C) Spp1 and the Type II marker, Calb2, label different subsets of HCs. (D) Quantification of expression for Calb2, Anxa4, Mapt and Spp1 by cell type in P64 utricles. Results indicate comparable specificity for Calb2, Anxa4 and Mapt in Type II HCs. In contrast, Spp1 is highly specific for Type I HCs. Values are mean±s.d. Scale bar in A″, same for all other panels: 20 µm.

Therefore, we sought to identify novel markers for each of the two clusters containing mature Type I and Type II cells by examining genes that were highly differentially expressed between those two clusters. Two genes that showed strong expression in the putative Type II cluster, were *Anxa4* (*Annexin A4*) and *Mapt* (*Microtubule Associated Protein)* (Fig. 3B). To determine whether these genes represent novel markers for Type II HCs, expression of protein products for both was compared with Myosin7A (Fig. 4A,B). Results indicated labeling of a subset of adult HCs with both anti-Anxa4 and anti-Mapt. Anxa4 is localized to cell and nuclear membranes while Mapt is present in the cellular cytoplasm. Orthogonal images indicate that cells positive for Anxa4 or Mapt have morphologies that are consistent with Type II HCs (Fig. 4A″,B″). The cell bodies are generally located close to the lumenal surface and the HCs do not have the narrow necks that characterize Type I HCs. To confirm that both Anxa4 and Mapt are specifically expressed in Type II HCs, utricles were double-labeled for the Type II marker, Calb2 and either Anxa4 or Mapt. Results indicated a broad overlap of expression between Calb2 and Anxa4 or Mapt (Fig. 4C,D). To determine the specificity and extent of expression for both Anxa4 and Mapt, the number of Type II HCs in P64 utricles that express either marker was quantified (Fig. 5C). Results indicated that Anxa4 and Mapt are expressed in most (greater than 65%) Type II HCs and in a very small number (6% or less) of Type I HCs. While the prevalence of either Anxa4 or Mapt is not as great as Calb2 (81% of Type II HCs), the specificity for Type II HCs is comparable (Fig. 5D).

One gene that showed strong, and almost exclusive, expression in the putative Type I HC cluster was *Secreted Phosphoprotein 1* (*Spp1*), previously referred to as Osteopontin (Fig. 3B). To determine which cell types in the adult utricle express Spp1, adult utricles were double-labeled with anti-Spp1 and anti-Myo7A. Results indicated double-labeling for Spp1 and a subset of Myo7A-positive HCs (Fig. 5A–A″). Moreover, orthogonal views indicated that HCs that express Spp1 have morphologies that are more consistent with Type I HCs, including a flask shape and a more basal nucleus (Fig. 5A″). This was also evident in whole mount views when focused at the level of Type II HC nuclei (Fig. 5′, inset). To confirm that Spp1^+^ HCs are Type Is, utricles were double-labeled with Spp1 and Tenascin C (Tnc). Tnc protein is enriched in the synaptic cleft between the calyceal endings surrounding Type Is, making it a suitable marker for this cell type (Lim et al., 2011; Lysakowski et al., 2011; Warchol and Speck, 2007). Consistent with a Type I localization, Spp1 and Tnc are co-localized in P70 utricle (Fig. 5B–B′). In the orthogonal view, Spp1 is clearly localized lumenal to Tnc in the neck region of each Type I cell. To confirm that Spp1 is confined to Type I HCs, utricles were double-labeled with anti-Spp1 and anti-Calb2. In contrast with the Type II marker Anxa4, there was no overlap between Calb2 and Spp1 (Fig. 5C). Finally, expression of Spp1 was quantified in P64 utricles based on HC morphology (Fig. 5C). Results indicate that nearly 88% of Type I HCs are positive for Spp1 as opposed to only 4.25% of Type II HCs. These results establish Spp1 as a novel pan marker of Type I HCs (both striolar and extrastriolar) and confirm that the second mature cluster observed in the single-cell data set represents Type I HCs. In addition, while striolar HCs are almost certainly a distinct subtype of HC, the combined results suggest that the difference between striolar and extrastriolar HCs is subtler than the region-independent difference between Type I and Type II.

### Developmental expression of Calb2, Anxa4, Mapt and Spp1

**Fig. 6.**
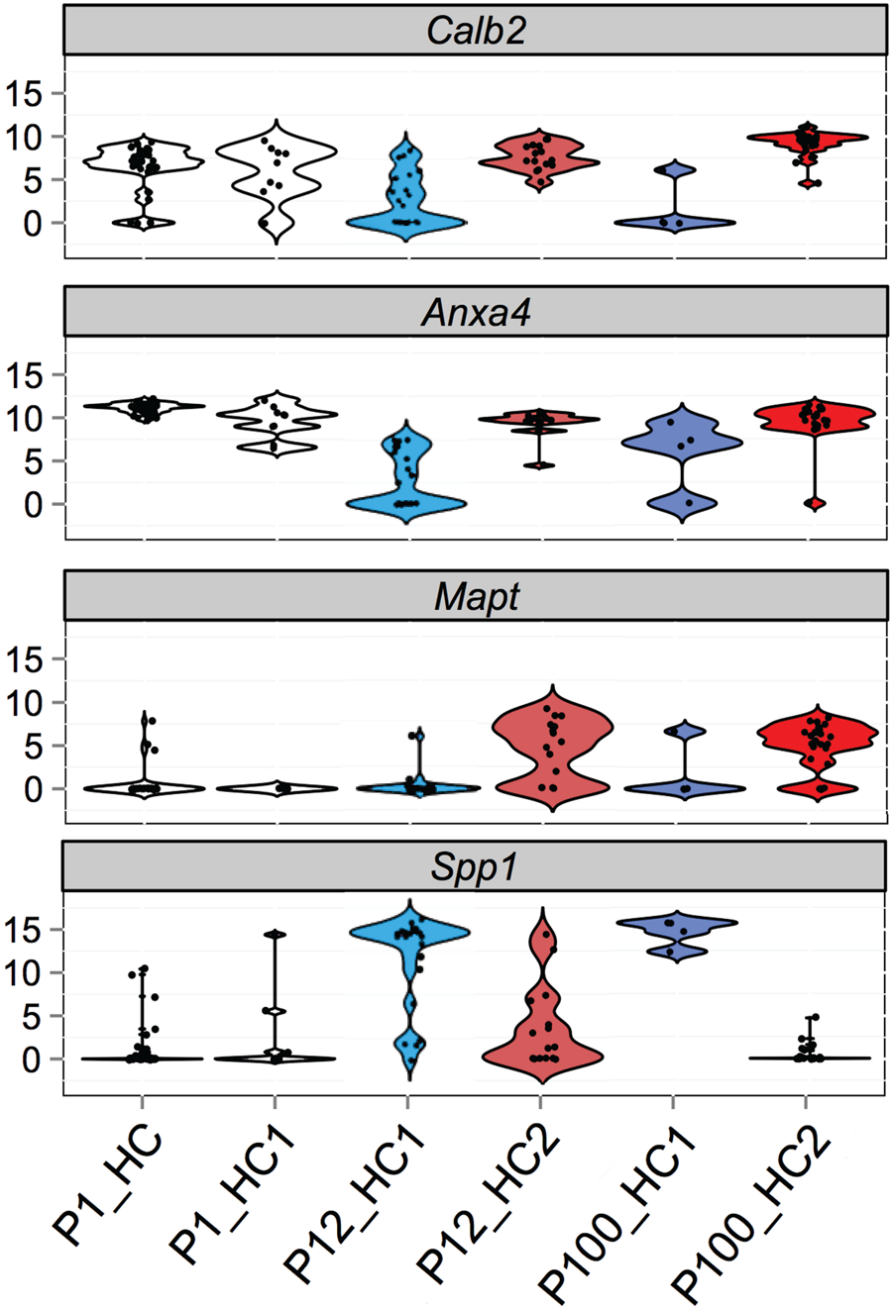
**Temporal expression data for HC type marker genes.** Violin plots based on scRNAseq data for the indicated genes in different HC types at different ages. At P1, *Calb2* and *Anxa4* are broadly expressed in all utricular HCs. In contrast, *Mapt* and *Spp1* are only detected in a small number of cells. At P12, *Calb2* and *Anxa4* expression is maintained in Type II HCs (HC2) but have been down-regulated in Type I HCs (HC1). At the same time point, *Mapt* shows a marked increase in HC2 while *Spp1* shows an increase in HC1. At P100, *Calb2*, *Anxa4* and *Mapt* all show strong expression in HC2 while *Spp1* and *Anxa4* are expressed in HC1. The number of HC1 cells that were positive for *Anxa4* is not consistent with the immunolocalization data which indicates restriction of Anxa4 to Type II HCs.

To determine when each of the novel markers becomes restricted to specific cell types, the single cell RNA-Seq data presented in Fig. 3 was separated based on age of collection. At P1, cells could be identified as either immature or Type I. No definitive Type II HCs were present. For P12 and P100, all cells could be identified as either Type I or Type II. As reported previously (Zheng and Gao, 1997), *Calb2* was uniformly expressed in virtually all utricular HCs at P1 (Fig. 6). By P12, expression of *Calb2* was significantly down-regulated in most Type I HCs but maintained at P1 levels in Type II HCs. At P100, the level of expression of *Calb2* was maintained in Type II HCs. In addition, a small number of Type I HCs also showed expression of *Calb2*. Whether these represent Type I cells that maintain expression throughout maturation or cells that re-express *Calb2* is unclear. Heat maps of gene expression did show a subset of adult cells co-expressing a number of Type I and II markers (Fig. 3A), suggesting some mature HCs may have a dual identity; however, these could also represent a technical difficulty in which two cells are captured in a single well on the microfluidics chip (see the Materials and Methods for further details). *Anxa4* showed a similar pattern of expression in that it was broadly expressed in both HC types at P1 (Fig. 6). However, in contrast with *Calb2*, many HCs, regardless of type, were still positive for *Anxa4* at P12. In fact, even at P64 when Anxa4 antibody labeling was mostly restricted to Type II HCs, some Type I HCs were still positive for mRNA for *Anxa4*. Despite the appreciable presence of transcripts in all clusters, differential expression of *Anxa4* was significant at P12 and P100 (*P*<0.05).

In contrast with *Calb2* and *Anxa4*, *Mapt* and *Spp1* were barely detectable in either HC cluster at P1. However, both demonstrated marked and specific increases in expression by P12 in Type II (*Mapt*) and Type I (*Spp1*) HCs, indicating that these genes distinguish mature HC subtypes from immature HCs.

**Fig. 7.**
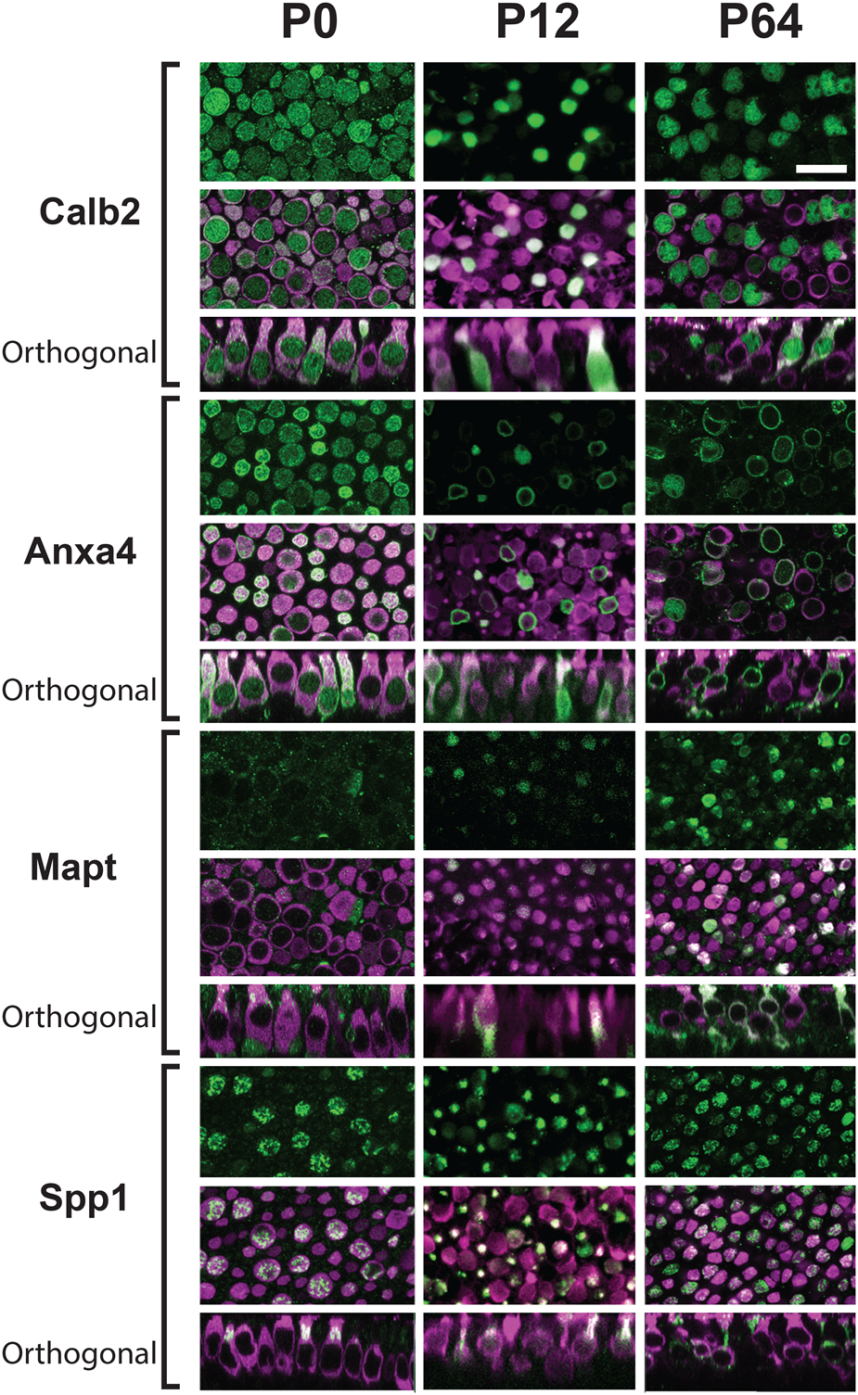
**Immunolocalization of HC type specific markers at different developmental time points.** Surface and orthogonal views of the indicated markers (green) and Myo7a (magenta) at the indicated time points. At P0, Calb2 and Anxa4 are broadly expressed in nearly all HCs. In contrast, Mapt is rarely expressed in HCs while Spp1 is expressed in only a limited number. The orthogonal view indicates that Spp1 is already restricted to the neck regions of HCs. At P12, Calb2 and Anxa4 are expressed in fewer HCs while expression of Mapt and Spp1 has increased. Finally, at P64, expression of Calb2, Anxa4 and Mapt is largely restricted to Type II HCs while Spp1 is restricted to Type I HCs. Scale bar: 10 μm.

To confirm the single cell RNA-seq results, immunolabeling for each marker was performed on utricles at P0, P12 and P64 and cell-type-specific labeling was then quantified based on HC morphology (Fig. 7; Fig. S1). At P0, Calb2 and Anxa4 were observed in 90.5% and 91.0% of all HCs, respectively. At P12 Calb2 expression was observed in over 80% of Type II HCs and in 30.3% of Type I HCs. Finally, at P64, expression of Calb2 in Type I HCs was maintained at 80% but only 3.5% of Type I HCs were positive. Anxa4 showed a similar pattern of expression with greater than 65% of Type II HCs positive at both P12 and P64, but with a decreasing expression in Type I HCs at both P12 and P64. Mapt was barely detected in cells in the P1 utricle but was observed in a significant number of Type II HCs at P12 and P64 (Fig. S1). Finally, Spp1 was expressed in a limited number of HCs at P1. In cells that could be definitively classified as Type I HCs, Spp1 was expressed in 60% while in hair cells that were classified as immature, only 23% were positive for Spp1. Interestingly, in either HC cell type, Spp1 was localized to the necks of cells (Fig. 7). At P12 the number of Spp1^+^ Type I HCs had increased to 90%, a level that was maintained at P64 as well. Expression in Type II HCs dropped from 20% at P12 to 4% at P64. Based on these results, expression of Mapt and Spp1 are markers of maturity that can be used to examine the development of Type II and Type I HCs respectively. These findings also support the idea that Type II HCs are a truly differentiated subtype distinct from an immature state. Finally, the results of the immunolabeling correlate well with the single cell data, providing validation for single cell analysis as a way to identify new markers for unique cell types.

### Temporal development of Type I and Type II HCs

**Fig. 8.**
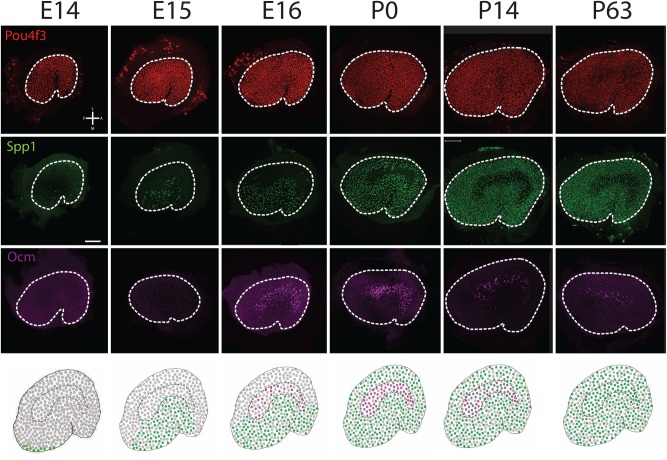
**Temporal and spatial development of Type I HCs.** Low magnification surface images of entire utricles obtained at the indicated time points. The upper three rows show a pan specific HC label (Pou4f3 in red), and the Type I HC markers Spp1 (green) or Ocm (magenta). The bottom row illustrates a cartoon summary of Spp1 (green) and Ocm (magenta) expression at each age. At E14, while the utricle contains a uniform distribution of HCs, Spp1^+^ HCs, which are predominantly Type Is, are restricted to the extreme medial-posterior region of the epithelium. At E15, Spp1^+^ cells have extended anteriorly and laterally. At E16, Spp1^+^ cells are present in the entire medial half of the utricle and Ocm^+^ HCs are present in the striola. At P0, cells that express Spp1 are present throughout the utricle with the exception of the striolar HCs which still express Ocm. At P14, Spp1^+^ Type I HCs are now present in the striola while expression of Ocm has decreased. At P63, Ocm^+^ HCs are almost completely absent, while most striolar Type I HCs are now positive for Spp1. Orientation for all images is indicated in the upper left hand panel. Scale bar: 100 µm.

The Atoh1-lineage tracing experiments described earlier in this study indicated that initial HC development within the utricle occurs in a central-to-medial gradient followed by a central-to-lateral gradient. To better understand whether development of specific cell types follows a similar pattern, embryonic and early post-natal utricles were labeled with Spp1 or Mapt antibodies to identify Type I and Type II HCs, respectively. While these markers are not exclusively expressed in one HC type or the other at early developmental time points, they do show a strong bias towards expression in Type Is (Spp1) or Type IIs (Mapt). In addition, since there is a delay in the onset of expression of Spp1 in striolar HCs (Fig. 8), utricles were also labeled with anti-Oncomodulin (Ocm), which has been shown to mark striolar Type I HCs starting at late embryonic time points (Hoffman et al., 2018; Simmons et al., 2010). Finally, utricles were counter stained with a pan-HC marker, either anti-Pou4f3 or anti-Myosin 7A based on species compatibility, to mark the full HC population.

**Fig. 9.**
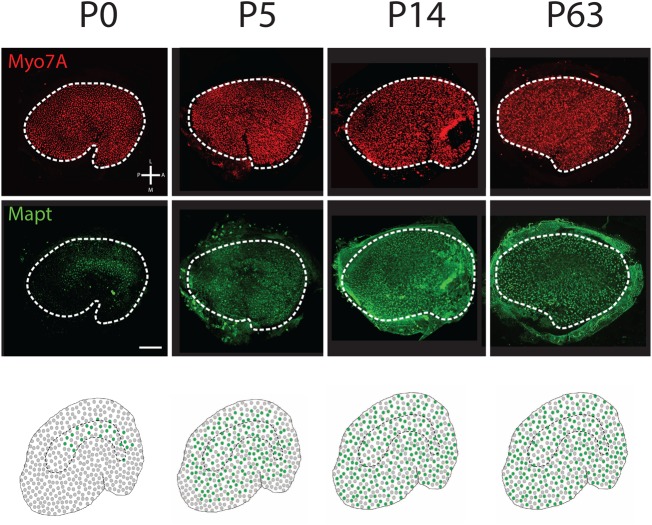
**Temporal and spatial development of Type II HCs.** Low magnification surface images of the entire utricle obtained at the indicated time points. The upper two rows show a pan specific HC label (Myo7A in red), and the Type II HC marker (Mapt in green). The third row presents cartoon summaries of the expression data in the upper panels at each age. Minimal expression of Mapt was observed prior to P0. At P0, Mapt^+^ cells are present in the central region of the utricle. At P5, Mapt^+^ Type II HCs are present throughout the utricle with the exception of the extreme periphery and at P14 and P63, Mapt^+^ cells have extended throughout the utricle. Orientation for all images is indicated in the upper left hand panel. Scale bar: 100 µm.

At E14, HCs are present across the entire sensory epithelium (Denman-Johnson and Forge, 1999) (Figs 8,9). However, only a small number of HCs located at the extreme medial edge of the utricular epithelium express Spp1 (Fig. 8). At E15 and E16, HCs have been added uniformly across the epithelium while Spp1^+^ HCs have expanded laterally from the medial edge. Spp1^+^ cells are not present in the striola, but the onset of expression of Oncomodulin in the striola at E15 is consistent with an ongoing lateral wave of Type I HC formation. By P0, the wave of Spp1^+^ expression has reached the lateral side of the utricle. At P14 some Spp1^+^ HCs are present in the striola and at P63 most striolar Type I HCs are positive for Spp1. The onset of Spp1 expression in striolar HCs correlates with a down-regulation of Ocm.

In contrast with Type I HCs, no Mapt^+^ HCs were observed in the utricle at E14, E15 or E16 (not shown). However, a small number of Mapt^+^ HCs were observed in the central region of the utricle at P0 (Fig. 9). At P5, Mapt^+^ HCs were present throughout most of the utricle with the exception of the extreme periphery and at P14 Mapt^+^ HCs were present throughout the utricle. Based on these results, HC types appear to develop in temporally and spatially distinct patterns with Type I HCs developing prenatally in a medial-to-lateral pattern while Type II HCs develop later, almost entirely post-natally, in a more isotropic central-to-peripheral pattern.

### Most Type I HCs are generated prior to birth

**Fig. 10.**
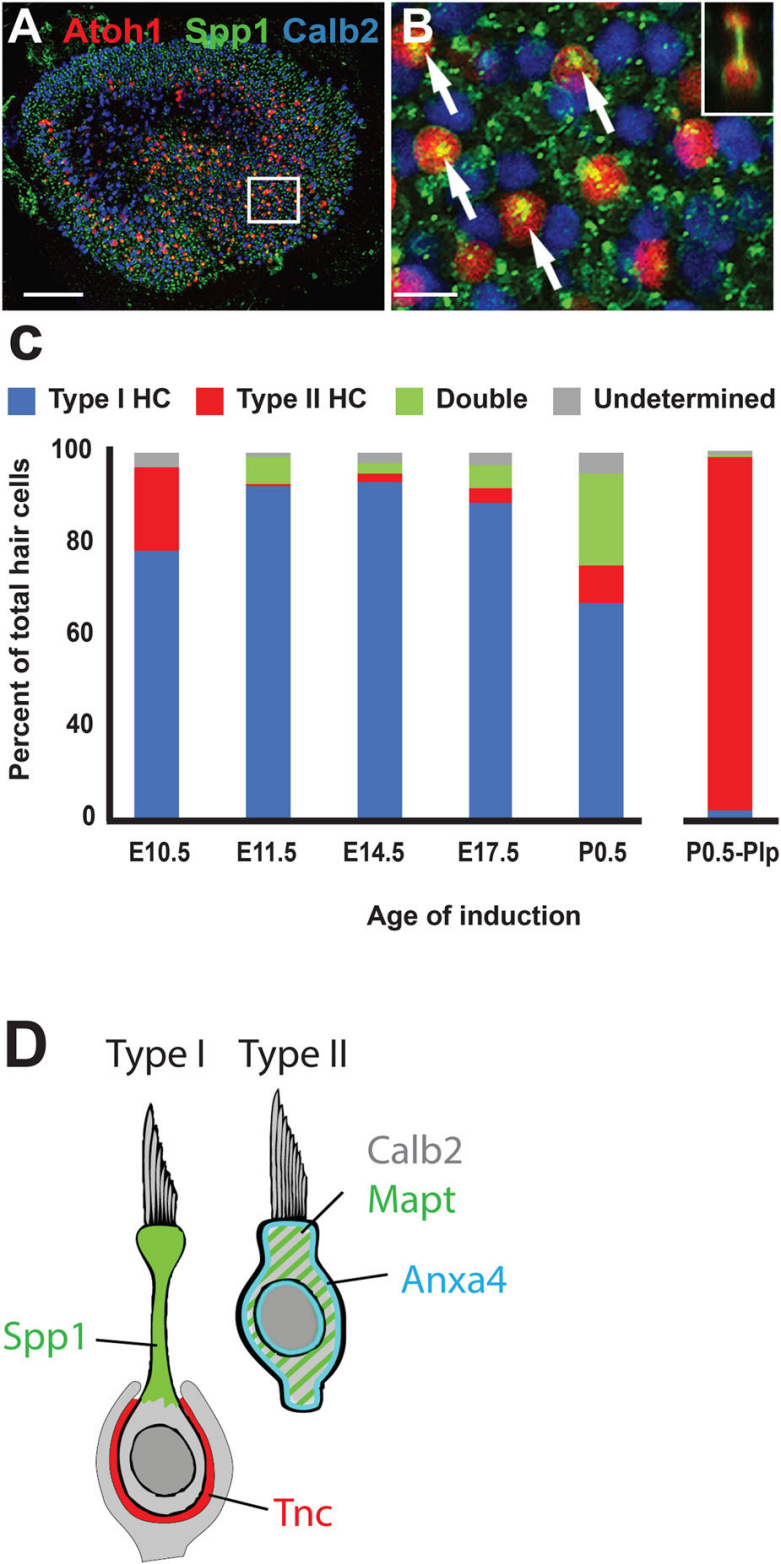
**Most Type I HCs are generated before birth.** (A) Low magnification image of a P75 utricle from an *Atoh1^creErt2^;R26R^tdtomato^* mouse induced at E14.5. *Atoh1^+^* cells are labeled in red. Type I HCs are labeled by expression of Spp1 (green) and Type II HCs are labeled by expression of Calb2 (blue). (B) High magnification view of the boxed region in A. Many of the red cells are also positive for expression of Spp1 (arrows). Inset: Orthogonal view for one of the cells in B. Note the expression of Spp1 in the neck region of this *Atoh1^+^* Type I HC. (C) Bar graph indicating the fates of *Atoh1^+^* or *Plp^+^* cells induced on the indicated days. Nearly all *Atoh^+^* cells generated prior to birth develop as Type I HCs. In contrast, nearly all *Plp^+^* progenitors labeled at P0.5 develop as Type II HCs. (D) Summary diagram illustrating cellular localization of Spp1 in the neck of Type I HCs, Mapt in the cytoplasm of Type II HCs and Anxa4 in the cell and nuclear membranes of Type II HCs. Scale bar in A: 100 µm, B: 10 µm.

The results presented in the previous section were consistent with previous results that suggested that Type I HCs are present at birth in the mouse while Type II HCs mature later (Rusch et al., 1998). To determine the temporal window for generation of HCs that will ultimately develop as Type Is, the *Atoh1^CreErt2^* line was used to mark presumed nascent HCs at E10.5, E11.5, E14.5, E17.5 or P0.5. Animals were then aged to at least P60 prior to being euthanized. Utricular HC types were determined based on expression of Spp1 as a Type I HC marker and Calb2 for Type II HCs. While not 100% specific for each cell type, Spp1 is expressed in 90% of all Type I HCs and less than 5% of Type II HCs while Calb2 shows a similar, but reciprocal expression pattern. Similar to the results presented in Fig. 2, the number of *Atoh1^+^* cells labeled at E10.5 was small, on average only about 10 per utricle, while inductions on E11.5 or E14.5 labeled several hundred cells (Table S1). Inductions on E17.5 or P0.5 labeled approximately 1000 cells per utricle (Table S1). Of the small number of nascent HCs labeled at E10.5, nearly 80% develop as Type Is (Fig. 10). The percentage of Type Is increases to approximately 90% at E11.5 and E14.5 and then shows a gradual decrease at E17.5 and P0.5 with only 60% of *Atoh1^+^* cells labeled at P0 developing as Type I HCs. To determine whether this trend continues into the post-natal period, *Plp1^creErt2^* was used to label a subset of the precursor/supporting cell population at P0 (Fig. S2). In contrast with *Atoh1*, which is transiently expressed at an early stage of HC development, *Plp1* is constitutively expressed in all progenitor/supporting cells. Therefore, induction of *Plp1^creErt2^* marks a subset of progenitors that will differentiate into HCs post-natally (Bucks et al., 2017; Burns et al., 2012), regardless of developmental state. Cell type analysis at P60 then allows a determination of the fates of progenitor cells between P0 and the day the mice were euthanized. As expected, the vast majority (87.48%) of *Plp1^+^* cells develop as supporting cells (Table S1). Of the *Plp1^+^* cells that do develop as HCs, only 2.85% develop as Type Is while nearly 96% develop as Type IIs. To confirm that labeling with *Plp1^creErt2^* reflects the accumulation of HCs over time, mice were induced at P0 and fixed on P3. The total number of Spp1^+^ or Calb2^+^ HCs was then compared between samples fixed on P3 versus P60. While the average number of *Plp1^+^* cells increased from 3464 at P3 to 3952 at P60 (1.14-fold change), the average number of *Plp1^+^* Type II HCs increased from 37 at P3 to 481 at P60 (13-fold change).

Finally, previous studies in the developing cochlea have demonstrated that only 70% of the cells that initially express *Atoh1* go on to develop as HCs, while the remaining 30% develop as supporting cells through notch-mediated lateral inhibition (Driver et al., 2013). To determine if a similar process occurs in the utricle, the number of *Atoh1^+^* cells that developed as HCs of any kind or supporting cells was examined. Results indicate that throughout the embryonic period most *Atoh1^+^* cells develop as HCs (Table S1). At E10.5, nearly 97% of *Atoh1^+^* cells develop as HCs; however, this ratio may be skewed because of the relatively small number of labeled cells at this age. Between E11.5 and P0.5, the percentage of *Atoh1^+^* cells that become HCs shows a relatively normal distribution that increases from approximately 70% at E11 to a peak of 94% at E14 and then declines to 84% by P0.

## DISCUSSION

The development and subsequent diversification of methodologies for the isolation and transcriptional profiling of single cells provides a powerful new approach for the identification of known and novel cell types within heterogeneous organs or tissues (Cuevas-Diaz Duran et al., 2017; Haque et al., 2017). In this study, the Fluidigm C1 platform was used to isolate 76 HCs from utricles at P12 and P100. The total number of cells analyzed is low by comparison with other available approaches for the isolation of single cells, but the C1 system allows for visual inspection of each captured cell prior to lysis as well as providing full length transcriptional data and, on average, greater coverage of the transcriptome. Moreover, using the C1 system to capture P12 and P100 utricular HCs allowed us to easily incorporate these data into our existing P1 data set (Burns et al., 2015). Despite the comparatively small number of cells analyzed, the results did identify several novel markers for both Type I and Type II HCs. In particular, as discussed below, Spp1 and Mapt should be particularly useful as they are cell-type specific in the adult and are not expressed in immature, probably more generic, HCs.

In contrast with the cochlea, the timing and spatial distribution of HC development in the utricle has not been studied extensively. The results of the Atoh1-fate mapping experiment are largely consistent with previous work in the rat indicating a general central-to-peripheral gradient of HC commitment and differentiation that mirrors the pattern of terminal mitosis (Sans and Chat, 1982). However, the earliest labeled *Atoh1^+^* cells do show a bias towards the medial half of the utricle, confirming that commitment occurs in a central-to-medial gradient followed by a central-to-lateral gradient (Burns et al., 2012; Denman-Johnson and Forge, 1999).

More intriguing is the temporal and spatial development of specific HC types. While immature HCs arise in a central-to-peripheral gradient beginning as early as E13 (Denman-Johnson and Forge, 1999), the earliest cell-type specific markers are not detectable prior to E14, at which time only two to three cells express the Type I marker Spp1. As development continues, expression of Type I markers indicates a gradient of cellular development that extends from medial-to-lateral between E14 and P0. In contrast, the Type II marker, Mapt, is not detectable prior to P0 and appears in a central-to-peripheral gradient. These results suggest a potential delay in the specification of mature utricular cell types. The basis for the differing patterns of development for Type Is versus Type IIs are unclear but could be related to the more derived nature of Type I HCs, which are only found in birds and mammals (Burns and Stone, 2017), suggesting that they represent a relatively novel HC type. Why they develop in a gradient that differs from the patterns for all immature HCs and Type IIs is unknown, but as the pattern extends along the medial-lateral axis, it is tempting to hypothesize a role for a morphogen or other gradient that might arise on, or adjacent to, the medial edge of the sensory patch.

The observation that Type I HCs develop almost exclusively during the embryonic time period while Type II HCs do not begin to express cell-type specific markers prior to P0 confirms previous physiological results suggesting that Type I HCs develop first and are present at birth (Geleoc et al., 2004; Nordemar, 1983; Rusch et al., 1998). However, it is interesting to note that while Spp1^+^ cells are present throughout the utricle at P0, the scRNAseq analysis classified only a limited number of HCs (5) isolated at P1 as Type Is. There are several possible explanations for this result. The first is simply that the proportion of Type I HCs at P1, by comparison with all HCs, is relatively low. A second possibility might be that variations in the sizes of different HC types could lead to a sampling error. The Fluidigm microfluidics chips are designed to capture cells of particular sizes. However, a third explanation might be that the maturation of Type I HCs is an extended process that could take as long as 14 days. Therefore, at P1, even though many cells have begun to develop as Type Is based on expression of Spp1, the transcriptional profiles of these cells are not sufficiently different from other HCs to be identified as a unique cell population based on unbiased clustering analysis.

Similarly, cluster analysis of HCs from P12 or P100 was only able to resolve the two primary HC types. However, anatomical and electrophysiological data suggests the existence of at least two types, striolar and extrastriolar, of both Type I and Type II HCs (Engstrom and Wersall, 1958; Desai et al., 2005; Li et al., 2008; Geleoc et al., 2004; Hoffman et al., 2018; Hurley et al., 2006; Schweizer et al., 2009; Simmons et al., 2010). While it is possible that functional differences are not reflected at a transcriptional level, it may also be the case that the limited number of cells analyzed for this study was insufficient to resolve differences within a particular cell type. With the recent development of higher through-put methods for the collection and analysis of single cells, it should be possible to collect a significantly greater number of utricular HCs in the future, which should provide greater resolution in terms of cell-type specific transcriptomes.

Three new cell-type specific markers, Anxa4 and Mapt for Type II HCs and Spp1 for Type I HCs, were identified and validated. Differential expression analysis also identified many other potential new markers, which were not validated here. A summary of the validated marker expression patterns is illustrated in Fig. 10D. While our primary interest in these genes was as new markers for vestibular HC types, it is reasonable to postulate about the roles of each of these genes in HC function. Anxa4, a member of the Annexin family of phospholipid binding proteins, has been shown to localize to cytoplasmic and nuclear membranes in response to elevated Ca^2+^ levels (Arii et al., 2015; Grewal et al., 2016; Heinick et al., 2015). Consistent with increased Ca^2+^ in HCs in response to transduction, cytoplasmic and nuclear localization of Anxa4 was observed in Type II HCs. The specific roles of Anxa4 are somewhat unclear, although other Annexins play a role in shuttling specific molecules to the plasma or nuclear membrane (Bandorowicz-Pikula et al., 2012; Domon et al., 2012). Interestingly, elevated Anxa4 levels have been correlated with resistance to platinum-based chemotherapy drugs (Matsuzaki et al., 2014; Morimoto et al., 2014), suggesting that at least some of the heterogeneity in vestibular HC death in response to cisplatin-treatment (Callejo et al., 2017; Cunningham and Brandon, 2006) could be a result of expression of Anxa4 in Type II HCs. Consistent with this hypothesis, Type II HCs show greater resistance by comparison with Type I HCs following cisplatin treatment *in vitro* (L.L. Cunningham, personal communication).

Spp1, formerly Osteopontin, is a non-collagenous osteogenic matrix protein that is present in otoconia and has been reported to be expressed in HCs of the utricle and saccule (Takemura et al., 1994; Uno et al., 2000). However, specific expression of Spp1 in Type I HCs had not been determined in the past. While Spp1 is prominent in mammalian otoconia and plays a role in bone formation in a number of systems, *Spp1* mutants have no obvious defects in otoconial formation and vestibular function is normal suggesting either that it is dispensable (Zhao et al., 2008), or that there may be functional compensation by another matrix protein, as has been demonstrated in *Oc90* mutants in which there is an increase in Sparcl1 (Xu et al., 2010).

Mapt (Tau) has been extensively studied as a microtubule-associated protein that plays a role in the elongation and stabilization of microtubules. In vestibular HCs, prominent microtubules are present in the kinocilia and in association with the cuticular plate where they serve to link the plate to the lateral cell membrane (Surel et al., 2016; Takumida et al., 1995; Vranceanu et al., 2012). However, immunolocalization indicated limited expression of Mapt in HCs prior to P5, suggesting a limited role in the initial development of either the kinocilia or the cuticular plate. Analysis of phenotypes in Mapt mutants suggest a stronger role in maintenance (Šimić et al., 2016), which is consistent with the timing of expression that we observed.

Finally, several lines of evidence have suggested that Type I HCs might transit through a Type II-like state during their development or that mature Type II HCs might ultimately transform into Type I HCs (Kirkegaard and Jørgensen, 2000; Masetto and Correia, 1997; Weisleder et al., 1995). The results of the fate-mapping and cell-type specific expression data presented here suggest that neither of these scenarios is correct. The Type II marker Mapt was not observed at any embryonic time points, even though virtually all Type I HCs develop pre-natally. Similarly, tracking the fates of early post-natal progenitors indicated that virtually all utricular HCs that develop post-natally are Type IIs. This observation is consistent with the lack of expression of Mapt prior to P0. What does appear clear from PCA analysis and overlap of markers between immature and mature cells is the Type II cells are relatively less distinct from the immature state compared to Type I HCs.

Overall these results suggest that generic, immature HCs develop during the embryonic time period in a medial-to-central followed by central-to-peripheral gradient that begins as early as E10.5. By E14.5, a subset of those immature HCs begins to develop as Type Is. However, rather than mirroring the initial HC development gradient, the first Type I cells are located at the extreme posterior-medial edge of the sensory epithelium. Type I development proceeds in a wave that extends towards the anteriorly and laterally across the utricle such that by P0 Type I HCs are present throughout the utricular epithelium. Expression of Type II specific markers are not observed prior to P0 and the pattern of their development largely mirrors the initial pattern of HC development.

## MATERIALS AND METHODS

### Animals and dissection of utricles

All experiments were conducted in accordance with the NIH Guide for the Care and Use of Laboratory Animals and were approved by the NINDS/NIDCD ACUC. CD-1 mice, obtained from Charles River Laboratories, were used as breeders and as wild-type mice for antibody labeling. *Atoh1^creErt2^* mice (Fujiyama et al., 2009) were kindly provided by Stephen Maricich, Case Western Reserve, and *Plp1^creErt2^* mice were provided by Brandon Cox, Southern Illinois University School of Medicine (Gómez-Casati et al., 2010; McGovern et al., 2016). The Tg(*Lfng-EGFP*)HM340Gsat BAC transgenic mouse line (*Lfng^EGFP^*) was generated by the GENSAT project (Gong et al., 2003) and was obtained from A. Doetzlhofer (Johns Hopkins University); B6;129S6-*Gt(ROSA)26Sor^tm9(CAG-tdTomato)Hze^*^/^ J mice (*R26R^CAG-tdTomato^*) were generated by H. Zeng and were obtained from Jackson Laboratories; and *Gfi1^Cre/+^* mice (*Gfi1^Cre^*) were generated (Hwang et al., 2010) and generously provided by L. Gan (University of Rochester). These three lines were crossed to generate mixed-background *Lfng^EGFP^*; *R26R^CAG-tdTomato^*; *Gfi1^Cre^* mice that express GFP in all inner ear sensory patches and tdTomato in HCs (Burns et al., 2015). Mice of either sex were used for all experiments. *Lfng^EGFP^*; *R26R^CAG-tdTomato^*; *Gfi1^Cre^*mice were used for single-cell RNA-Seq experiments at the indicated ages.

To perform lineage tracing, a single dose of tamoxifen was administered to pregnant females on embryonic day 10 (E10.5), E11.5, E14.5, or E17.5 (100 ml/mouse at a concentration 20 mg/ml, oral gavage). Tamoxifen was also administered to *Atoh1^creErt2^*; *R26R^tdTomato^* or *Plp1^creErt2^*;*R26R^tdTomato2^* pups through IP injection at post-natal day 0 (P0.5) or P1.5. Pups were then raised to maturity (>P60) prior to euthanasia and dissection of utricular maculae.

Briefly, inner ears were dissected out of the temporal bone and utricles were isolated. The utricular roof was dissected away and otoconia were removed using an eyelash prior to fixation in fresh 4% paraformaldehyde in phosphate-buffered saline (PBS) at room temperature for 1 h. Both right and left utricles were used and sex of the animals was not noted.

### Dissociation and single-cell capture

Single-cell dissociation and capture was performed as described previously (Burns et al., 2015). Briefly, for each utricular cell capture, four to five *Lfng^EGFP^*; *R26R^CAG-tdTomato^*; *Gfi1^Cre^* mice from one to three litters were euthanized at P12 or P100. At P12 and P100 in these mice, GFP expression is restricted to extrastriolar supporting cells. tdTomato is expressed at high levels in all HCs and at low levels in an undetermined cell population within the underlying mesenchyme. The vestibular labyrinth was removed from each ear (*n*=8–10 utricles per single cell capture), and utricles were isolated in ice-cold DMEM/F-12 (Life Technologies). The roof and non-sensory epithelium were trimmed away with forceps, and otoconia were removed with a hair. A small strip of transitional epithelium at the border between sensory and non-sensory epithelium was included to be sure that all cells within the sensory epithelium were isolated. The presence of GFP and tdTomato signal was verified with a fluorescence stereomicroscope, and utricles were transferred to a solution of DMEM/F-12 containing 0.2 mg/ml thermolysin (Sigma-Aldrich), 10 units/ml elastase (Worthington) and 10 kunitz/ml DNase I (Stem Cell Technologies) for 30 min at 37°C. The organs were then returned to ice-cold DMEM/F-12 where the epithelium was separated from the underlying mesenchyme, removing any non-HCs that might express tdTomato. The delaminated epithelia were collected in a curette and transferred to a 1.5 ml Lo-Bind Eppendorf tube containing 20 units/ml papain, 1 mM L-cysteine, 0.5 mM EDTA, 10 kunitz/ml DNase I in 0.5 ml of EBSS. The tube was placed with the cap open in an incubator at 37°C and 5% CO_2_ for 1 h, and during this period the tube was removed every 15 min to gently triturate the epithelia with a 200 μl pipette. After the incubation, the dissociated cells were gently triturated 10–20 times with a blunted 26-G needle.

At the end of the incubation period, papain was inhibited by adding an equal volume of 10% ovomucoid in DMEM/F-12. The suspension was then sequentially passed through a 20 µm and 10 µm strainer (Pluriselect) to remove clumps of cells, and the dissociated cells were then pelleted at 300 ***g*** in a swinging bucket centrifuge for 5 min at 4°C. The supernatant was aspirated until only 15–20 μl remained, and the cells were resuspended and placed on ice until counting and capture.

Cell capture, lysis, SMARTer-based RT and PCR amplification of cDNA was performed on a Fluidigm C1 platform as outlined in the manufacturer's protocol (PN 100-7168 I1 and in Burns et al., 2015). Briefly, after obtaining a single-cell suspension, 10 μl of cells at a final concentration of 2.5×10^5^–7×10^5^ cells per ml were loaded onto a medium-sized (10–17 μm) integrated fluidics circuit (IFC). Cell concentration was estimated at a 1:10 dilution using an automated cell counter (Luna). The IFC was placed in the C1 system, where cells were automatically washed and captured. After capture, the chip was removed from the C1, and a 30 μm stack of widefield fluorescence and brightfield images was recorded at each capture site using a ×10/0.4 numerical aperture objective on an inverted Zeiss Axio Observer.Z1 microscope equipped with a motorized stage. A custom script was written within the Zeiss Zen Blue software to automate this process. Average imaging time for all 96 capture sites was 35 min. After the imaging period, the IFC was returned to the C1 where lysis, RT and PCR were performed automatically within individual reaction chambers for each cell. For RNA-Seq, mixes were prepared from the SMARTer Ultra Low RNA kit (Clontech) according to the volumes indicated in the Fluidigm protocol. The thermal cycler within the C1 performs 21 rounds of PCR amplification to obtain enough material for RNA-Seq. cDNA was manually collected from the output channel of each capture site and stored in a 96-well plate at −20°C until library preparation. The average time from dissection to cell lysis was ∼3 h. Cells from P12 mice were captured on two IFCs in two independent experiments, and cells from P100 mice were captured on three IFCs in three independent experiments.

### Single-cell RNA-Seq

Single cells were selected from the z-stack images of each capture site, eliminating any sites with suspected multi-cell or empty captures. Fluorescent protein levels were not used to select cells in this study, but were instead used to confirm expected identity from the unbiased clustering analysis. Therefore, a mix of GFP-positive, tdTomato-positive, and negative cells were prepared for sequencing.

The concentration of cDNA obtained from each capture site was measured in duplicate using a PicoGreen assay (Life Technologies) and a Beckman Coulter DTX 880 fluorescence plate reader. cDNA was diluted to a final concentration of 0.1–0.3 ng μl^−1^, then tagmented and tagged with adapter sequences using the Nextera XT DNA Sample Preparation Kit (Illumina), as described in the Fluidigm protocol. The adapter sequences were then used as primer recognition sites for a limited-cycle PCR reaction (12 cycles) in which sequencing primer and unique barcode sequences were added using the Nextera XT DNA Sample Preparation Index Kit (Illumina). Finally, barcoded libraries from 48 cells were pooled and cleaned using AmPure XP beads (Agencourt). Single-cell libraries originating from multiple C1 captures were pooled together to avoid compounding any potential C1 capture bias with sequencing lane bias.

Each collection of 48 pooled single-cell libraries was sequenced on a single-flow cell lane of an Illumina HiSeq 1000 to an average depth of 3.4 M reads using 90×90 paired-end reads. Reads were de-multiplexed and then aligned to a Bowtie index based on the NCBI-annotated mouse transcriptome (extracted from the 26,583 genes in GRCm38 genome with the corresponding GTF) using Bowtie 2v2.2.3. The sequences and identifiers for enhanced GFP (EGFP) and tdTomato were appended to the genome FASTA and the GTF before creating the index used for alignment. For each cell (library), relative transcript abundances were estimated from the aligned reads using RSEM v1.2.19 (default parameters). RSEM estimates transcript abundance in units of transcript per million (TPM). The abundances reported here are at the gene level, which RSEM calculates by summing the estimated transcript abundances for each gene. Alignment and abundance estimation were carried out on the NIH/Helix Biowulf cluster. TPMs from the P12 and P100 samples were combined with a previously published dataset of 158 cells from the P1 mouse utricle (Burns et al.). Relative transcript abundance estimates were normalized by scaling each sample by the median of the geometric means across all samples.

### Analysis of scRNAseq data

Any cells that appeared unhealthy in the recorded capture site images were excluded from library preparation. To further identify potentially unhealthy cells with abnormally low expression levels, we passed the cells through the outlier identification function provided in SINGuLAR Analysis Toolset 3.0, Fluidigm's R package for single-cell expression analysis. Outlier identification in SINGuLAR proceeds by trimming low-expressing genes until 95% of the genes that remain are above 1 nTPM in half of the cells. A distribution of combined gene expression is created from these cells, and outliers are considered as cells whose median expression across the identified gene list is below the 15th percentile of the distribution. Using these routines, 15 cells were excluded from the analysis. The resultant dataset consisted of 330 cells spanning the three ages.

To identify the HCs within the dataset, we employed hierarchical clustering, which appeared to segregate HCs from supporting cells and transitional epithelium cells at the first node on the resultant dendrogram. The cluster contained all of the HCs previously identified as such in the published P1 dataset, and every cell except for one was tdTomato-positive in the capture site images. Based on this evidence we selected the 113 cells from the HC cluster for further analysis. The distribution by age was 37 P1 cells, 51 P12 cells, and 25 P100 cells. The supporting cell and transitional epithelial cell data is not the subject of this report and will be presented elsewhere.

To identify subtypes of HCs, unbiased clustering was performed with Seurat v2.0 (Satija et al., 2015). Briefly, genes with detectable expression in 2 or fewer cells were removed from the dataset, which resulted in a data matrix of 15,560 genes by 113 cells. The gene by cell matrix was then log normalized, scaled, and centered. Principal component analysis (PCA) was performed using a subset of genes with high variance, and the resultant PCA was projected onto the full gene set. For unbiased classification of cells, Seurat uses a shared nearest neighbor algorithm on a pre-defined number of PCs. We chose the first four PCs based on a PCA elbow plot and set the clustering resolution to 1. This method identified three unique clusters in the dataset, consisting of immature, Type I, and Type II HCs, as described in the Results. Tests of differential expression were performed in Seurat with default parameters.

### Immunohistochemistry

Utricles were permeabilized and blocked for 2–3 h at room temperature in 0.5% PBS-Triton (PBS-T) and 10% normal donkey or goat serum (Vector Labs). Samples were incubated overnight at 4°C in primary antibodies diluted in 2% donkey/goat serum and PBS-T, followed by three rinses in PBS-T. Primary antibody labeling was detected using Alexa Fluor-conjugated secondary antibodies (1:400; Invitrogen) and/or DAPI (1:1000) also in 2% donkey/goat serum, PBS-T for 3 h. Finally, samples were washed three times with PBS and mounted in SlowFade (Life Technologies) for imaging. Images were taken using a Zeiss LSM 710 microscope at 40×/1.4 Oil DIC M27 and/or 20×/0.8 M27. The following primary antibodies and dilutions were used in this study; Ms α Calbindin2 at 1:200 (MAB1568; Millipore), Rb α Calbindin2 at 1:200 (AB5054; Millipore), Gt α Anxa4 at 1:200 (AF4146; R&D Systems), Ms α Pou4f3 at 1:200 (sc-81980; Santa Cruz Biotechnology), Rb α Myosin VIIA at 1:200 (25-6790; Proteus BioScience), Gt α Spp1 at 1:200 (AF808; R&D Systems), Gt α Ocomodulin at 1:200 (sc-7446; Santa Cruz Biotechnology), Rb α Tnc at 1:200 (AB19013; Millipore), Ms α Mapt at 1:200 (4019; Cell Signaling Technology).

### Quantification of marker expression

Utricles obtained at P0, P12 or P64 were labeled using antibodies for Myosin7a, as a pan hair cell marker, and one or more of the following, anti-Calb2, Anxa4, Mapt or Spp1. Whole mounts were imaged on a Zeiss LSM 710 using expression of Myosin7a to identify hair cells. Hair cells were classified as Type I or Type II using the ortho function in Zen Black. Then expression of individual markers was determined. A minimum of three samples were counted for each time point and sample with a minimum of 80 cells examined per sample.

### Quantification of cell fates

In utricles from *Atoh1^creErt2^*;*R26R^tdTomato^* or *Plp1^creErt2^*;*R26R^tdTomato2^* animals induced at E10.5, E11.5, E14.5, E17.5, P0.5, or P1.5, all cells positive for tdTomato were counted manually using the Cell Counter plug-in of ImageJ (NIH). Cells were determined to be HCs or supporting cells based on morphological differences between the two; supporting cell nuclei were found to be lower in the sensory epithelium and appeared star-like at their apical surface, this method of identification was confirmed in some (but not all) samples where supporting cells did not appear to be labeled by generic HCs markers, Myo7a or Pou4f3. tdTomato^+^ HCs were determined to be Type I or Type II based on the presence or absence of Opn (Type I) and/or Calb2 (Type II) and on morphology. Cells that showed restricted expression of Spp1 in a neck region were counted at Type Is. Cells that were negative for Spp1 but positive for Calb2 and had a non-flask shaped morphology were considered as Type IIs. Cells that expressed both Spp1 and Calb2 were counted as non-definitive. Tallies were also kept of HCs that were found to contain both Type I and Type II labels, Opn and Calb2, or contained neither label.

## Supplementary Material

Supplementary information
