# Supplementary material for: Characterization of spatial and temporal development of Type I and Type II hair cells in the mouse utricle using new cell-type-specific markers
Source: Biol Open. 2018 Nov 15;7(11):bio038083. doi: 10.1242/bio.038083 (PMC6262869; doi:10.1242/bio.038083)
Supplement: Supplementary information [file biolopen-7-038083-s1.pdf]

Biology Open (2018): doi:10.1242/bio.038083: Supplementary information

Supplemental Table 1. Lineage tracing for Atoh1+ and Plp+ utricular hair cells. Upper panel indicates average number of each cell type that was positive for tdTomato and for the indicated cell type markers. Lower panel presents the same data but as a percentage of the total number of tdTomato+ cells. Blue box indicates percentage of labeled cells that develop as hair cells or supporting cells.

Cre-Driver	Sample Size (n)	Age at Induct.	Type I HC Spp1+	Type II HC Calb2+	Doubl. Pos. HC Spp1+/Calb2+	Unlabl. HC	Supp. Cell	Total tdTom+ HCs	Total tdTom+ Cells
Atoh1	4	E10	6	0	1.8	0.3	0.3	8	8.3
Atoh1	6	E11	103.3	8.8	0.7	1	50.7	113.8	164.5
Atoh1	7	E14	251.3	8.3	12	10	14.7	281.7	296.3
Atoh1	6	E17	931	76	44.6	35.6	159	1087.2	1246.2
Atoh1	6	P0	545.2	236.8	83	47.7	202.3	912.7	1115
Plp	6	P0	14.3	481	2.8	3.2	3451.3	501.3	3952.7

Cre-Driver	Age at Induct.	Type I HC Spp1+	Type II HC Calb2+	Doubl. Pos. HC Spp1+/Calb2+	Unlabl. HC	Total HCs	Total SCs
Atoh1	E10	75.71%	0.00%	20.71%	3.57%	96.88%	3.13%
Atoh1	E11	91.19%	7.42%	0.63%	0.76%	69.81%	30.19%
Atoh1	E14	88.74%	3.52%	4.45%	3.29%	94.61%	5.39%
Atoh1	E17	84.57%	7.58%	4.95%	2.90%	86.65%	13.35%
Atoh1	P0	59.77%	24.96%	9.99%	5.28%	84.50%	15.50%
Plp	P0	2.88%	95.67%	0.67%	0.78%	12.52%	87.48%

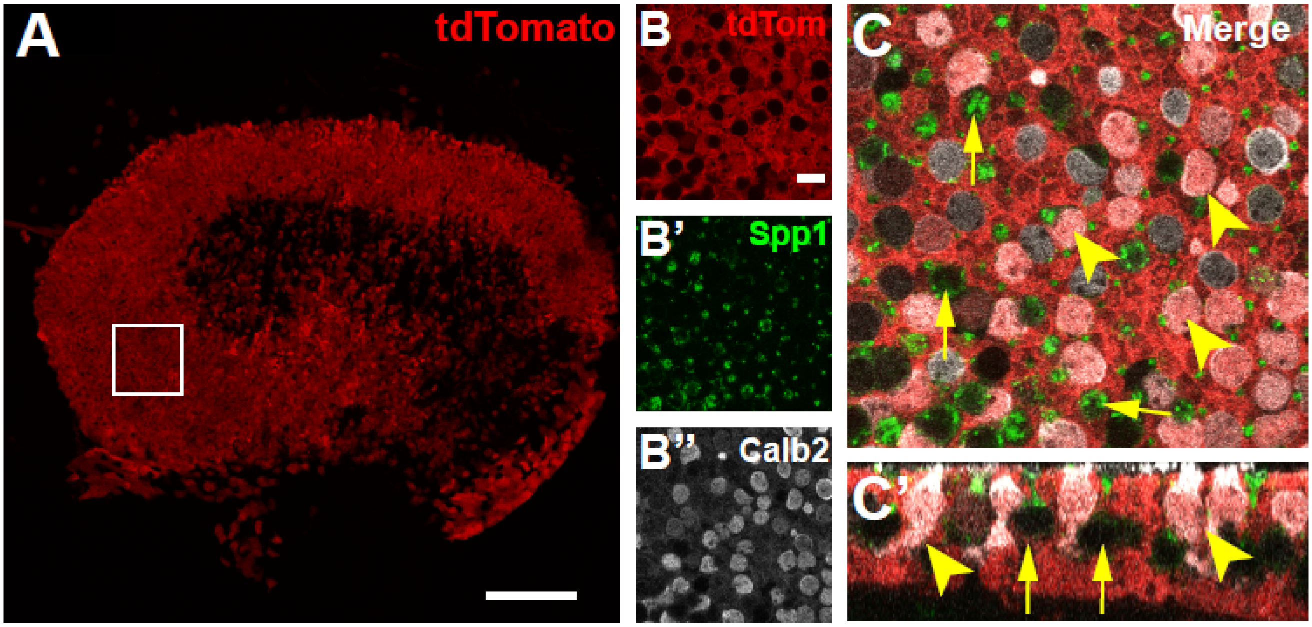

Supplemental Figure 1. Quantification of expression of hair cell markers. Average expression, as a percent of total hair cells counted, of the indicated markers at the indicated time points. Calb2 and Anxa4 are broadly expressed in all hair cells at P0. At P12 and P64, expression is maintained in Type II HCs but decreases in Type I HCs. In contrast, Mapt is only present in 6% of HCs at P0 but is observed in greater than 75% of Type II HCs at P12 and P64. AT P0, Spp1 is expressed in approximately 25% of all hair cells but in greater than 60% of those cells that can be identified at Type I HCs. At P12 and P64 approximately 90% of all Type I HCs are positive for Spp1 while the number of Type II HCs that express Spp1 drops to less than 5% at P64. The data presented for the P64 time point is also presented in Fig. 5D.

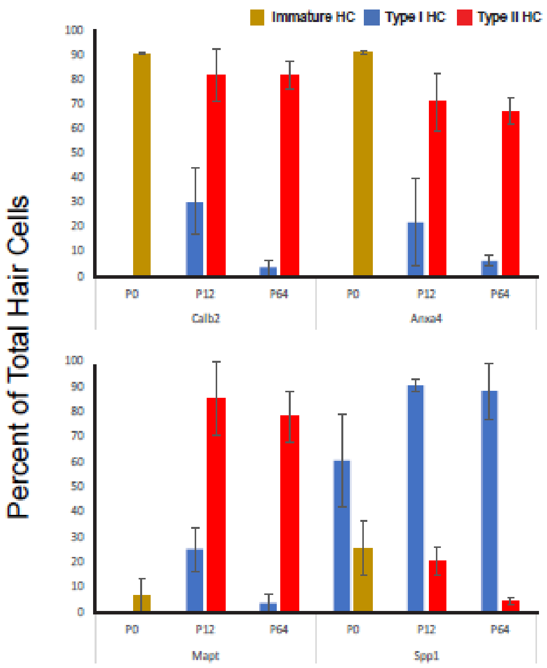

Supplemental Figure 2. Type I and type II HCs are generated postnatally. A. Low magnification image of an adult Plpcre;R26RtdTomato utricle induced on P1. Recombination (red) is uniform across the epithelium with the exception of the striolar region. B-B". High magnification view of the boxed region in A, illustrating expression of tdTom (B), the Type I HC marker Spp1 (B') and the Type II HC marker Calb2 (B"). Note the presence of tdTomato-negative cells in B, which most likely represent HCs that were generated prior to induction. C. Merged image of the panels shown in B. Most of the tdTomato negative cells appear to be Type I HCs based expression of Spp1 in the necks of those cells (arrows in C and C'). In contrast, many of the Calb2-positive Type I HCs are also positive for tdTomato (arrowheads in C and C'), indicating that they were generated postnatally.

Biology Open • Supplementary information
